# Supplementary figures and images for: Biochemical fractionation of human α-Synuclein in a Drosophila model of synucleinopathies
Source: bioRxiv. 2024 Feb 13:2024.02.05.579034. Preprint. [Version 2] doi: 10.1101/2024.02.05.579034 (PMC10871193; doi:10.1101/2024.02.05.579034)

Figure S1

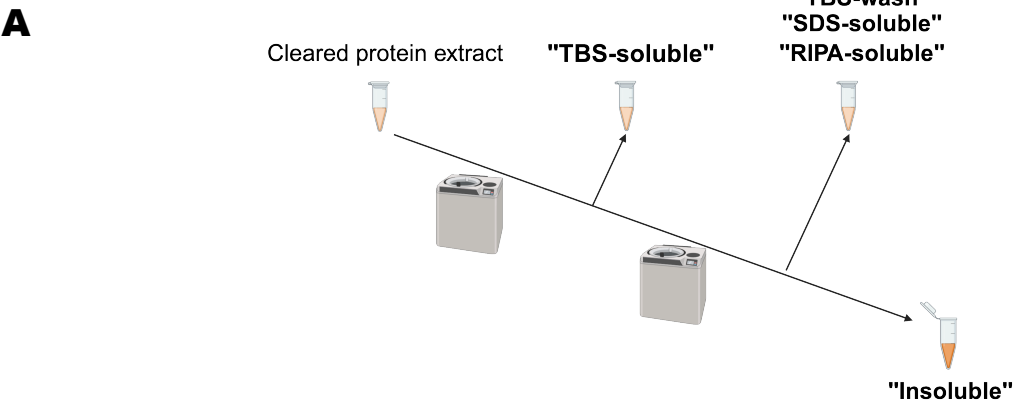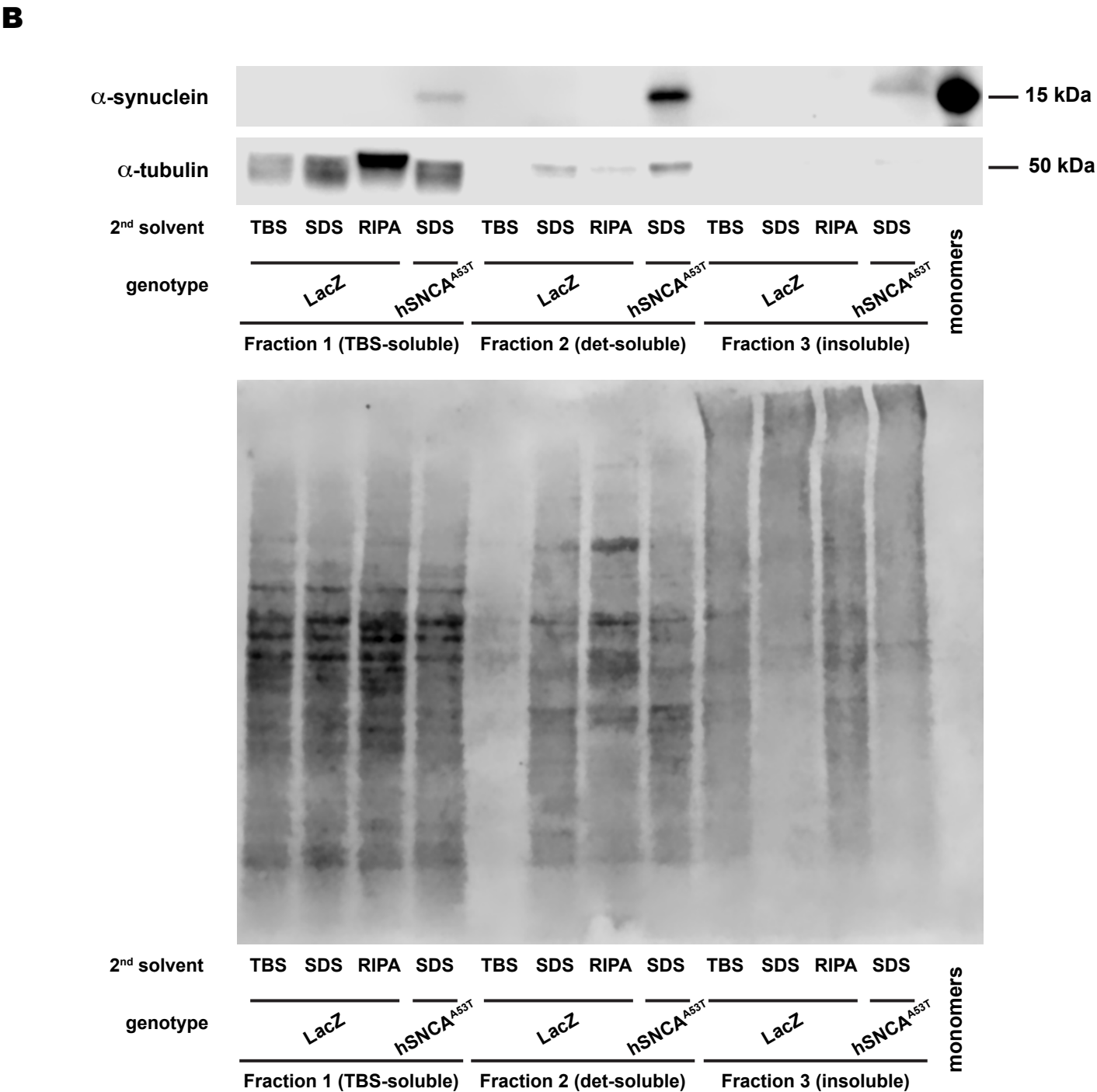

Supplement: Supplement 1 — (A) Schematic representation of the sequential fractionation protocol and the extraction buffers employed in this experiment. (B) Representative western blot of head lysates from flies expressing LacZ or hSNCAA53T in dopaminergic neurons. Fly heads are fractionated using a 3-step protocol in which the second fraction uses a variable detergent solvent, TBS, SDS or RIPA buffer. The first fraction (TBS-soluble) is loaded in lanes 1–4, second fraction (TBS-wash, SDS-soluble or RIPA-soluble) is loaded in lanes 5–8, and the third fraction (insoluble) is loaded in lanes 9–12, while 2ng of purified recombinant human α-synuclein monomers (monomer) are loaded in lane 13 as positive control. Protein lysates are extracted from flies expressing LacZ in dopaminergic neurons (w; +/+; TH-Gal4/ UAS-LacZ, lanes 1–3, 5–7, 9–11) and flies expressing hSNCAA53T (w; +/+; TH-Gal4/ UAS-hSNCAA53T, lanes 4, 8, 12). The fractions are probed for α-synuclein (4B12, top panel) and α-tubulin (T6074, bottom panel). Note that control flies (w; +/+; TH-Gal4/ UAS-LacZ) do not show any reactivity to the α-synuclein antibody regardless of the fractionation protocol. (C) Total protein staining with Revert Total Protein Stain of the membrane employed in this experiment. [file media-1.pdf]

Figure S2

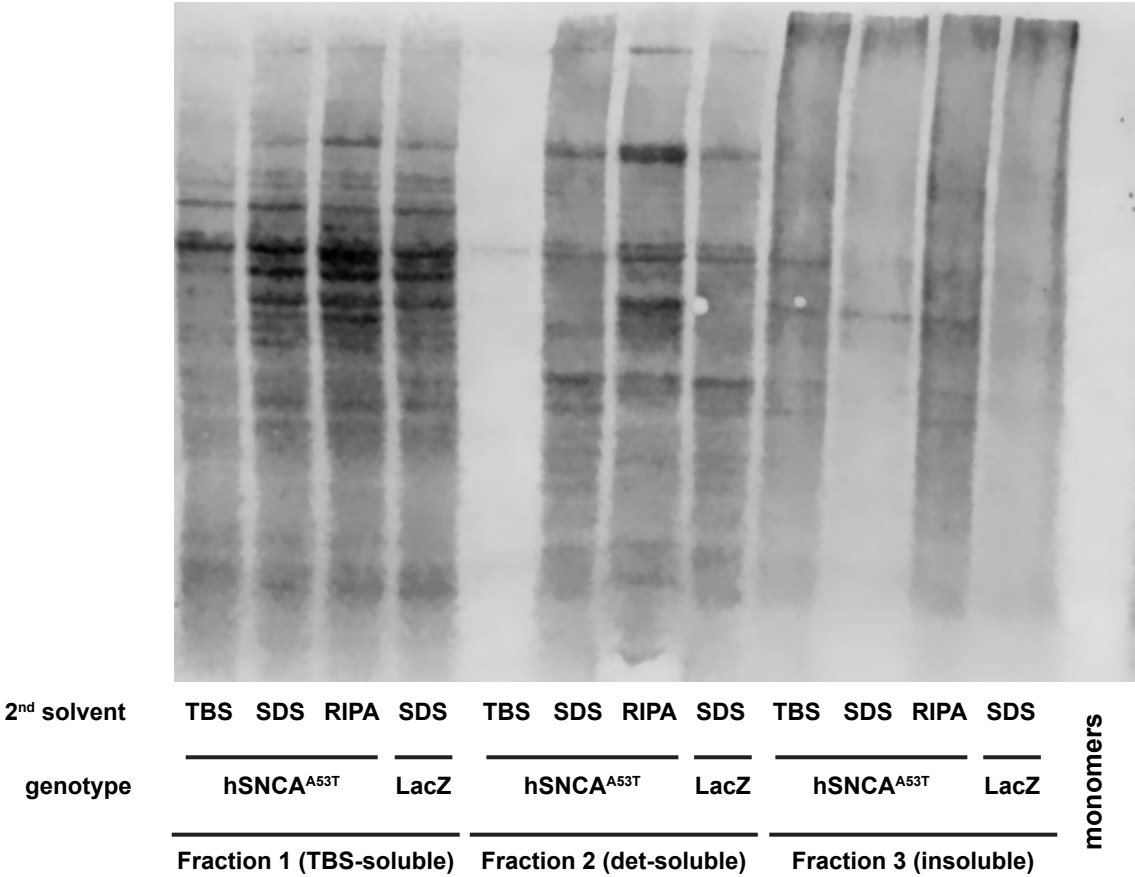

Supplement: Supplement 2 — Total protein staining with Revert Total Protein Stain of the membrane employed for the experiment in figure 2. [file media-2.pdf]

**Figure S3**

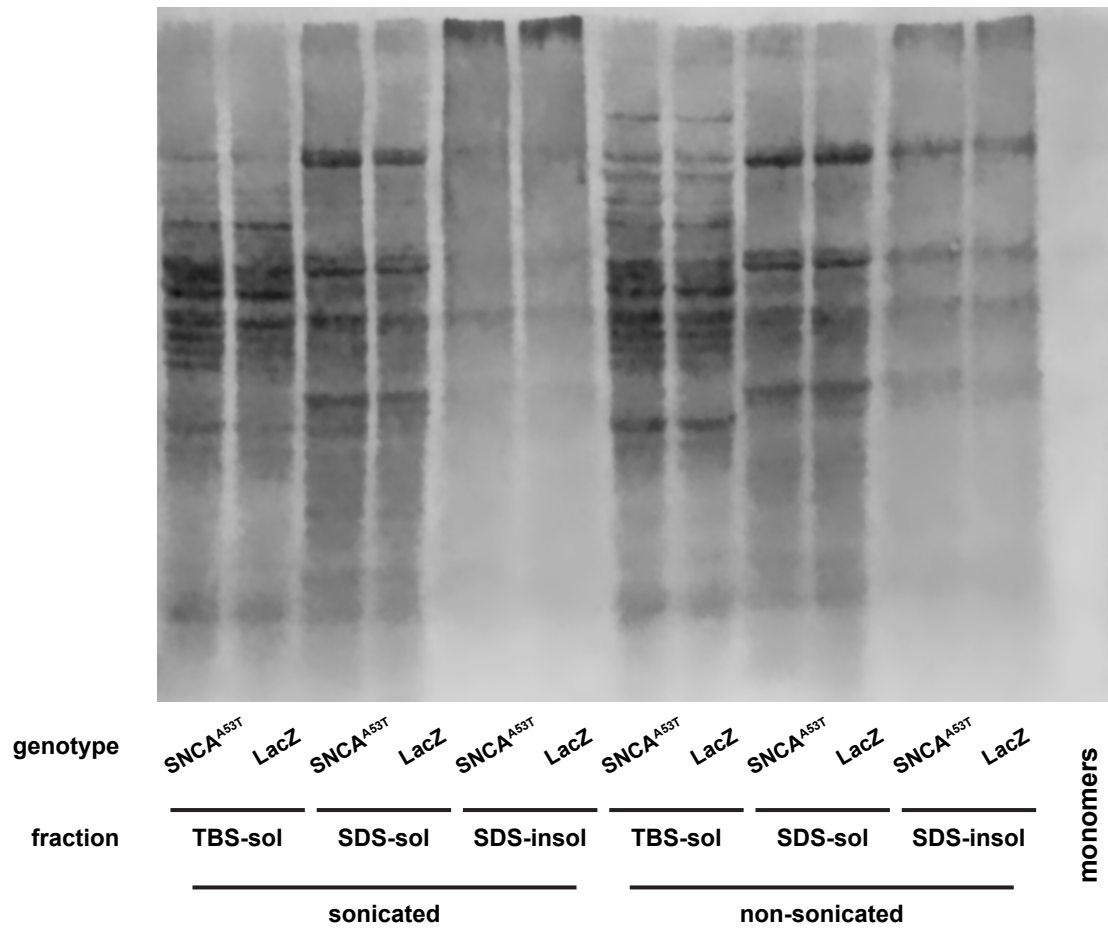

Supplement: Supplement 3 — Total protein staining with Revert Total Protein Stain of the membrane employed for the experiment in figure 3. [file media-3.pdf]

Figure S4

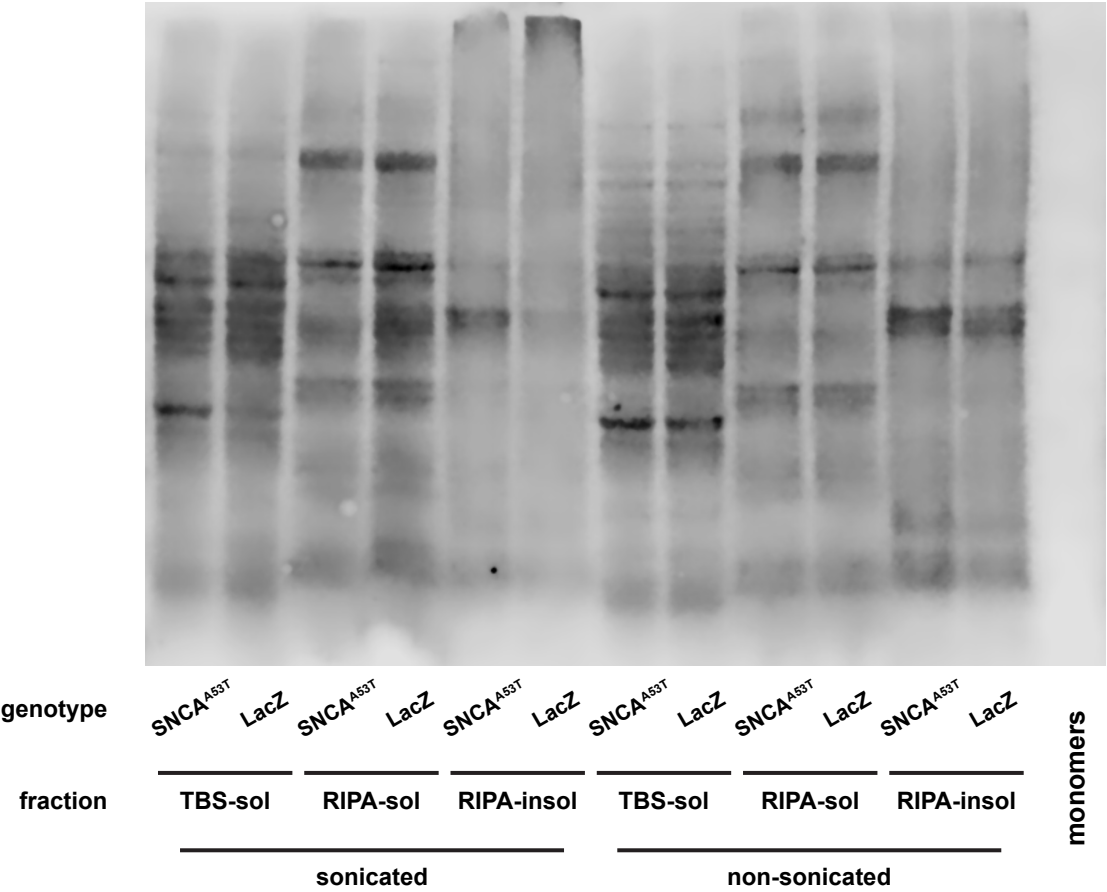

Supplement: Supplement 4 — Total protein staining with Revert Total Protein Stain of the membrane employed for the experiment in figure 4. [file media-4.pdf]

Figure S5

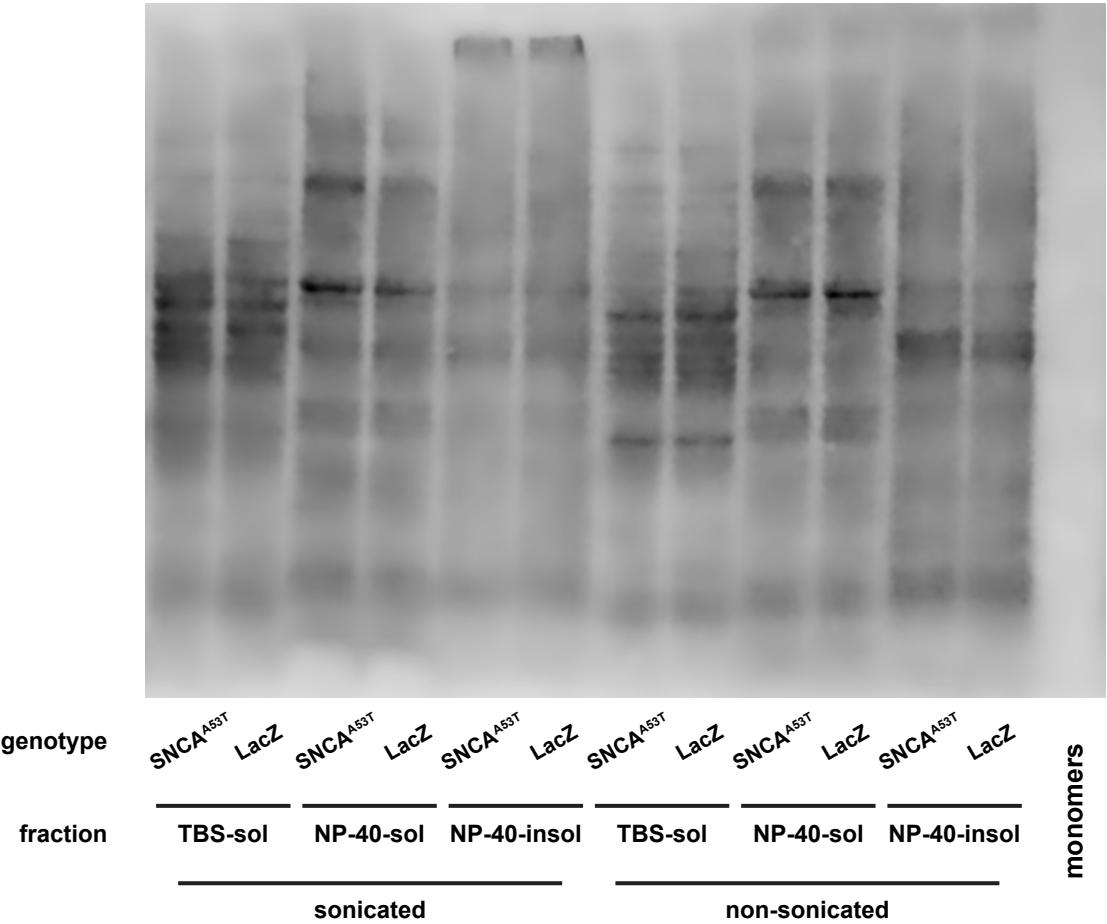

Supplement: Supplement 5 — Total protein staining with Revert Total Protein Stain of the membrane employed for the experiment in figure 5. [file media-5.pdf]

**Figure S6**

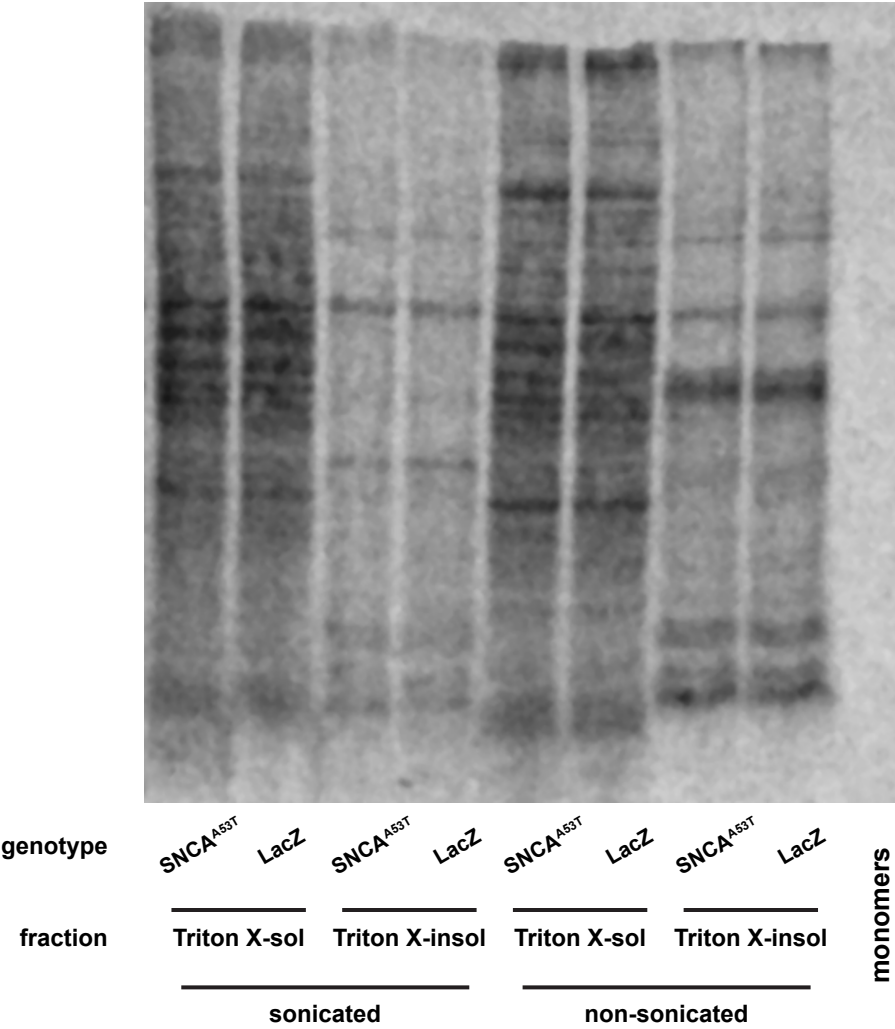

Supplement: Supplement 6 — Total protein staining with Revert Total Protein Stain of the membrane employed for the experiment in figure 6. [file media-6.pdf]
