# Supplementary material for: Biochemical fractionation of human α-Synuclein in a Drosophila model of synucleinopathies
Source: bioRxiv. 2024 Feb 13:2024.02.05.579034. Preprint. [Version 2] doi: 10.1101/2024.02.05.579034 (PMC10871193; doi:10.1101/2024.02.05.579034)
Supplement: Supplement 7 — Table S1. Comparison of α-synuclein enrichment in different fractions. The data show differences in mean values ± SEM of normalized α-synuclein signal detected in each biochemical fraction from TH-Gal4/UAS-hSNCAA53T flies (sonicated and non-sonicated data were grouped for this analysis), and p-values from post-hoc Tukey’s multiple comparisons assessing the significance of the differences between the compared groups. Normalization was performed in reference to total protein staining. Signal values are in arbitrary units (a.u.). Table S2. Comparison of α-tubulin enrichment in different fractions. The data show differences in mean values ± SEM of normalized α-tubulin signal detected in each biochemical fraction from TH-Gal4/UAS-hSNCAA53T flies (sonicated and non-sonicated data were grouped for this analysis), and p-values from post-hoc Tukey’s multiple comparisons assessing the significance of the differences between the compared groups. Normalization was performed in reference to total protein staining. Signal values are in arbitrary units (a.u.). [file NIHPP2024.02.05.579034v2-supplement-7.pdf]

bioRxiv preprint doi: https://doi.org/10.1101/2024.02.05.579034; this version posted February 13, 2024. The copyright holder for this preprint (which was not certified by peer review) is the author/funder, who has granted bioRxiv a license to display the preprint in perpetuity. It is made available under aCC-BY 4.0 International license.

Table S1. Comparison of α-synuclein enrichment in different fractions.

**Fractionation Sequence**	**Fractions compared** (fraction A – fraction B)	**Difference** (fraction A – fraction B)	**p-value**
**TBS>SDS>insol**	TBS - SDS	-1.119 ± 0.353	0.0120
	TBS - Insol	-1.059 ± 0.353	0.0176
	SDS - Insol	0.060 ± 0.364	0.9849
**TBS>RIPA>insol**	TBS - RIPA	-3.068 ± 0.614	0.0002
	TBS - Insol	0.429 ± 0.614	0.7676
	RIPA - Insol	3.496 ± 0.614	<0.0001
**TBS>NP-40>insol**	TBS - NP-40	-1.019 ± 0.284	0.0071
	TBS - Insol	0.730 ± 0.284	0.0527
	NP-40 - Insol	1.749 ± 0.284	<0.0001
**Triton>insol**	TritonX - Insol	1.834 ± 0.1253	<0.0001

Table S2. Comparison of α-tubulin enrichment in different fractions.

**Fractionation Sequence**	**Fractions compared** (fraction A – fraction B)	**Difference** (fraction A – fraction B)	**p-value**
**TBS>SDS>insol**	TBS - SDS	2.209 ± 0.227	<0.0001
	TBS - Insol	2.284 ± 0.227	<0.0001
	SDS - Insol	0.075 ± 0.227	0.9418
**TBS>RIPA>insol**	TBS - RIPA	2.081 ± 0.206	<0.0001
	TBS - Insol	2.144 ± 0.206	<0.0001
	RIPA - Insol	0.063 ± 0.206	0.9493
**TBS>NP-40>insol**	TBS - NP-40	3.338 ± 0.577	0.0001
	TBS - Insol	3.271 ± 0.577	0.0001
	NP-40 - Insol	-0.067 ± 0.577	0.9925
**Triton>insol**	TritonX - Insol	2.173 ± 0.216	<0.0001
